# Genetic Variants Influencing Taste Perception and Food Preferences: Current Evidence

**DOI:** 10.3390/nu18152531

**Published:** 2026-08-04

**Authors:** Ewa Miller-Kasprzak, Paweł Bogdański

**Affiliations:** Department of Treatment of Obesity, Metabolic Disorders and Clinical Dietetics, Poznan University of Medical Sciences, 61-701 Poznan, Poland

**Keywords:** taste preferences, taste perception, food-related behavior, single-nucleotide variants

## Abstract

Taste perception, taste preferences, and food preferences are three interrelated constructs underlying food-related behavior and contribute to individual dietary choices. Both genetic and environmental factors contribute to shaping these traits. Taste perception refers to interindividual differences in biological sensitivity to basic taste modalities. Taste preferences represent the hedonic evaluation of taste modalities, including salty, sweet, bitter, sour, or umami, and fatty tastes, and are closely related to, but distinct from, taste perception. Recent evidence in sensory genetics has identified numerous single-nucleotide variants (SNVs) associated with interindividual differences in both taste perception and taste-modality-related responses. Essential genes include *TAS1R2* and *TAS1R3* for sweet taste, *TAS1R1* and *TAS1R3* for umami, *TAS2R38* for bitter taste, *CD36* for fat perception, and epithelial sodium channel (ENaC) subunits for salt sensitivity. Regarding broader food preferences, variants in genes involved in metabolic regulation and reward processing—such as *FTO*, *MC4R*, *GHRL*, *DRD2*, and *OPRM1*—also contribute to food-related behaviors and choice patterns. Health status and genetic variants may also influence taste perception and food preferences. This review summarizes current evidence on the genetic determinants of taste perception, taste modality preferences, and food liking, emphasizing the multifactorial nature of food-related behaviors and the role of genetic variability in shaping individual differences in dietary choices.

## 1. Introduction

Taste, called gustation, is a sensory modality generated by the activation of taste receptor cells within oral taste buds [1,2,3,4]. It traditionally comprises five primary qualities: sweet, bitter, salty, sour, and umami. Additionally, other modalities, such as fatty acid taste (oleogustus) and metallic taste sense, have been frequently proposed as new taste quality candidates [1,4,5,6,7]. In this review, we focus mainly on genes involved in the gustatory system; however, it is important to emphasize that taste sensations during food consumption are far more complex than the gustatory system alone. The perception of food is broader and is referred to as flavor, which includes olfactory and somatosensory inputs, such as chemesthetic and trigeminal signaling. Thus, the terms “taste” and “flavor” are sometimes used interchangeably, although they describe distinct concepts [1]. The olfactory receptor family drives the olfactory system, and chemosensory modalities such as chilly burning or menthol cooling are mediated by the transient receptor potential (TRP) family. Trigeminal sensations are mediated by the sensory endings of the trigeminal nerve, namely the fifth cranial [8,9]. TRP family members are ion channels responsible for sensing a variety of stimuli, from vision and taste to heat and cold, to pain and stress responses [9,10]. Seven subfamilies have been established: TRP ankyrin (TRPA), TRP canonical (TRPC), TRP melastatin (TRPM), TRP mucolipin (TRPML), TRP NO-mechano-potential (TRPN, NOMP), TRP polycystin (TRPP), and TRP vanilloid (TRPV) [9,11]. A member of the polycystin subfamily, polycystic kidney disease 2-like 1 (PKD2L1), has previously been proposed as a direct sour-stimulus-perceiving taste receptor candidate [1,4,12]. The TRP family has been reviewed extensively [9,10]. Flavor perception also encompasses the olfactory system. Olfactory receptor proteins are expressed on olfactory receptor cells, and odorants bind to distinct receptor sites to transduce a signal of smell sensation [13]. When food is placed in the mouth, odorants go to the back of the throat and act on the olfactory epithelium, causing retronasal olfaction, which allows for the perception of flavor sensations [14]. A recent study confirmed that the insula serves as an integration hub for taste and odor patterns [14]. This interaction is important for food reward [14]. Food reward refers to the hedonic and motivational value of consumed food, encompassing both the pleasure derived from eating “liking” and the motivation to obtain and consume food “wanting” [15]. The dopaminergic circuit in the reward system influences food-related behavior and interacts with metabolic modulators to regulate the hedonic system [16].

In this review, we distinguish three constructs of food-related traits containing taste perception, taste preferences, and food preferences. Importantly, we used these terms in a specific operational sense, thus the terminology in the literature is not fully consistent. Taste preferences reflect affective responses to taste stimuli and may be influenced by both sensory and reward-related mechanisms [15,17]. There is increasing evidence that variants in genes involved in both the reward system and metabolic pathways may also be associated with taste and food preferences, and that broad genetic predispositions, such as to diet-related diseases, may affect dietary patterns [18].

Nutritional behavior is closely linked to health status and contributes to the development of numerous civilization-related diseases, including metabolic disorders. Conversely, health status may also influence food preferences and eating behavior. It has been documented that taste perception and food liking can change under various pathological conditions [19,20]. Moreover, genetic variants have been shown to influence food-related behaviors, particularly in the context of health and disease [21,22]. However, the biological mechanisms underlying these associations remain poorly understood, partly because the available evidence is derived predominantly from observational studies, which do not allow causal relationships to be established.

To identify relevant genetic association studies, a literature search was conducted across the PubMed/MEDLINE database. The search focused on peer-reviewed articles published up to July 2026, using combinations of the following keywords and Medical Subject Headings (MeSH): “taste perception”, “taste preference”, “food preference”, “genetic polymorphism”, “SNP”, “genetic variants”, “genome-wide association study”, “GWAS”, “TAS2R38”, “TAS1R”, and related taste receptor variants and non-taste receptors genes. The inclusion criteria primarily targeted original human studies examining the links between genetic variability and taste- or food-related phenotypes, including both large-scale cohort screens and targeted candidate-gene approaches. Non-English articles, abstract-only publications, and studies focusing strictly on animal models without human translation were excluded. While this manuscript represents a narrative review, this transparent strategy was applied to ensure a representative synthesis of the available evidence. Importantly, the genetic associations synthesized across the literature should not be interpreted as definitive causal relationships; genetic effects on taste and dietary behavior are generally modest and remain heavily modulated by age, cultural exposures, and overall health status.

This review focuses on the genetic variants of taste receptors and genes involved in metabolic and reward pathways, which may contribute to interindividual variability in taste perception and food-related behavior.

## 2. Three Interrelated Constructs Underlying Food-Related Behavior

In our model, taste perception refers to the sensory perception and intensity detection of basic taste modalities, typically measured using threshold or intensity-based methods. Taste preferences represent a hedonic evaluation (liking/disliking) of taste modalities and are operationalized using psychometric rating scales. Food preferences refer to the preferences for the consumption of specific food items or categories characterized by dominant taste qualities, e.g., particular cruciferous vegetables, fruits, salty snacks, sweet, bitter, or sour beverages.

We acknowledge that these terms are sometimes used interchangeably in the literature, thus we adopt a strict operational separation to improve clarity. To ensure this conceptual distinction is maintained without overinterpretation, specific phenotypes are strictly mapped onto these three constructs. Within this synthesis, taste perception accounts for biological taste sensitivity, while taste preferences isolate the hedonic element of liking. Broader behavioral and psychological expressions reported in the cited studies—including acute food intake, cravings, dietary habits, and consumption patterns—are systematically categorized under the broader domain of food preferences. These constructs are partially overlapping, related but not identical, and each is influenced by genetic factors, environmental exposures (including upbringing and lifestyle), and health status. In this context, it is worth noting that a broad range of genetic and environmental factors, along with a person’s health status, may interact in a multidirectional manner. Partial overlap shows that, e.g., someone who is more sensitive to bitter-sensing may at the same time prefer drinking coffee despite perceiving a stronger taste. In another example, a person who likes a salty taste may not prefer the intake of salty snacks due to restrictions caused by their health status. According to Drewnowski et al., highly preferred foods may be liked for reasons other than taste, whereas dislike or rejection reactions to food are much more strongly related to taste factors [17,23]. Some food preferences may depend on cultural behaviors rather than on true taste preferences [24].

Despite inconsistencies in methodological approaches and terminology across studies investigating associations between genetic polymorphisms and taste preferences, meaningful synthesis and interpretation of available evidence remain possible.

### 2.1. Most Common Methods of Assessing Taste Modalities

Taste detection is typically evaluated using psychophysical methods. Commonly used measures include detection thresholds (DTs), recognition thresholds (RTs), suprathreshold intensity ratings of prototypical tastant compounds, 6-n-propylthiouracil (PROP) bitterness intensity, and the number of fungiform papillae [25]. Both DT and RT indicate the lowest concentration of a particular tastant that can be detected and properly recognized by an individual, respectively. Suprathreshold intensity ratings assess perceived taste intensity at concentrations above the recognition threshold and provide information on the magnitude of sensory perception [25]. Additional evaluation methods include psychometric scales, such as the visual analog scale (VAS), category rating scales, and general labeled magnitude scales (gLMS), which are commonly used to evaluate taste intensity, pleasantness, and hedonic response across different taste modalities.

In contrast to taste function, taste preferences reflect affective evaluations of taste stimuli, such as liking or disliking particular taste qualities. Taste preferences are assessed using hedonic rating scales, preference ranking tasks, or food-preference questionnaires that evaluate the degree of liking for specific foods or taste categories. Food intake and habitual dietary patterns are typically assessed using dietary assessment tools, including food frequency questionnaires (FFQs), food diaries, and 24-h dietary recalls [26,27].

Together, these methods enable the investigation of taste perception, taste preferences, and food preferences [17,25,28].

### 2.2. Role of Taste Perception in Nutrition

The gustatory system plays a fundamental role in guiding food-related behavior. Acts as a biological link between the external chemical environment and the internal metabolic needs. By detecting potential beneficial or harmful compounds, it contributes to the regulation of food intake and maintains metabolic homeostasis [1,4,17,24,29]. A bitter taste warns us to avoid potentially harmful foods, and salt informs us about the source of required minerals and helps maintain water homeostasis. Sweets guide us to energy-rich foods and are hedonic; a sour taste signals the presence of acids and may help detect unripe, spoiled, or fermented foods. Umami (Japanese umami means “delicious”) guides the intake of peptides and proteins [29].

These processes are mediated by specialized taste receptor cells and taste receptors [1,2,3,4,29,30,31].

## 3. Biological and Functional Characteristics of Taste Receptors

Taste buds are functional gustatory organs composed of multiple cell types, including Type I, Type II, Type III, and basal cells [2,4,32]. Taste buds are found on various types of lingual papillae and are also present in the epithelium of the palate [1,2,4,33]. Taste receptors involved in the transmission of various taste stimuli represent different functional types of proteins. Depending on the taste modality signal transmitted, some are classical transmembrane G-protein-coupled receptors (GPCR), while others are plasma membrane ion channels and translocases/transporters [1,2,4,34,35]. Type I cells appear to function as glial-like cells. Type II cells are a source of GPCRs that detect sweet, umami, and bitter tastes; type III cells are involved in sour taste detection [1,2,4,33,36]. Recent studies have expanded the classical classification of taste bud cells, demonstrating that individual cell populations exhibit greater functional diversity than previously recognized. Type I cells may be engaged in the elimination of neurotransmitters and the redistribution and spatial buffering of K^+^. They have also been proposed to exhibit ionic currents linked with salt taste transduction. However, this population of bud cells appears heterogeneous, and its function remains a topic of ongoing debate, as does the identity of the primary sodium-responsive taste cell population [4,31,32,33,36]. Type III taste receptor cells are specialized for sour taste detection and form conventional synaptic connections with gustatory afferent fibers. These cells express proteins involved in proton detection and downstream neurotransmitter release and may respond to high salt concentrations [3,4,31,36].

The sensation of taste is experienced after activation of a particular taste receptor by a specific ligand (a tastant) and through the cellular signaling pathway transmitted to the gustatory cortex in the brain along the afferent sensory fibers [2,4,32,33].

### 3.1. Sweet, Umami, and Bitter Taste Receptors

Taste receptors for sweet, umami, and bitter modalities belong to the large family of GPCRs. The family consists of receptors that respond to a variety of processes, including smell, vision, and taste [2,37]. Type II cells in taste buds express receptors that recognize sweet and umami taste as well as receptors that transmit bitter taste stimulus. Both taste 1 receptor member 2 (TAS1R2) and taste 1 receptor member 3 (TAS1R3) subunits form a heterodimer responsible for sweet taste perception. In contrast, the heterodimer of taste 1 receptor member 1 (TAS1R1)/TAS1R3 mediates umami sense perception. Bitter taste is detected by monomeric receptors belonging to the large family of taste 2 receptors (T2R), among which taste 2 receptor member 38 (TAS2R38) is the most extensively studied and analyzed [1,2,38]. Signal transduction of sweet, bitter, and umami stimuli involves the G-protein α-gustducin, encoded by the G protein alpha subunit transducing 3 (*GNAT3*) gene. Notably, *GNAT3* is located in close proximity to the glycoprotein cluster of differentiation 36 (*CD36*) gene [39], which is implicated in fat taste perception. Although GPCRs are often considered to be expressed on distinct cell populations, the co-expression of TAS1R1, TAS1R2, and TAS1R3 has also been reported, suggesting that some taste receptor cells may respond to both sweet and umami stimuli [1,2,40]. Binding of appropriate ligands to heterodimeric sweet (TAS1R2/TAS1R3) or umami (TAS1R1/TAS1R3) receptors, or monomeric bitter receptors (TAS2R family), activates G protein α-gustducin and the Gβγ complex. The released complex activates phospholipase C β2 (PLCβ2) and leads to the production of inositol 1,4,5-trisphosphate (IP3) and diacylglycerol (DAG). The second messenger IP3 binds to IP3 receptors on the endoplasmic reticulum (ER), inducing Ca^2+^ release into the cytoplasm. Increased intracellular Ca^2+^ activates the TRPM4/5 complex, depolarizing the taste cell membrane and activating voltage-gated sodium channels (VGSC). This depolarization and increased Ca^2+^ triggers ATP release through calcium homeostasis modulator 1 (CALHM1/3) and pannexin channels. Extracellular ATP then activates purinergic receptors (P2X3/P2X3) on afferent gustatory nerve fibers, generating action potentials that are transmitted to the gustatory cortex for taste perception [1,2,4,36].

### 3.2. Salty Taste Receptors

Amiloride sensitive epithelial sodium channel (ENaC) is believed to contribute to salt taste transduction, particularly at low sodium concentrations [1,4,33,36]. The channel is composed of three main subunits, namely αβγ, each encoded by a separate gene: sodium channel epithelial 1 subunit alpha (*SCNN1A*), sodium channel epithelial 1 subunit beta (*SCNN1B*), and sodium channel epithelial 1 subunit gamma (*SCNN1G*). Salt taste transduction appears to involve distinct mechanisms depending on sodium concentration. Low-salt detection has been associated with an amiloride-sensitive ENaC-mediated pathway; however, the cellular localization and functional contribution of ENaC in taste buds remain unresolved. Although ENaCα, ENaCβ, and ENaCγ form a heterotrimeric sodium channel in other tissues, these subunits may not be co-expressed within the same taste cell populations, raising questions about the contribution of the canonical ENaC complex to sodium taste perception [36,41]. In contrast, high-salt perception is largely amiloride-insensitive and may involve other taste cell populations, including Type II and Type III cells. High-salt detection may not be restricted to taste buds. High concentrations of sodium may activate TRPV1 channels expressed on free trigeminal nerve endings in the oral mucosa, suggesting that somatosensory pathways also contribute to oral salt perception [33]. However, both the transduction mechanisms underlying sodium chloride (NaCl) transduction and the specific cells in taste buds responsible for transducing the signal remain incompletely elucidated [1,4,31,33,36,42]. In the current proposed model of perceiving salt taste stimuli, the influx of Na^+^ through amiloride-sensitive ENACα depolarizes the taste cell membrane. An additional Na^+^ influx generates action potentials by activating VGSC. This process ultimately triggers ATP release through CALHM1/3 channels, but the role of ATP also remains to be elucidated [31,33,36].

### 3.3. Sour Taste Receptors

Type III taste receptor cells in the taste bud transduce sour stimuli by allowing protons to enter the cytosol through a channel protein [2,3,4,33,36]. Notably, Type III cells have been found to respond to other tastants, including pure water, high-salt solutions, and carbonated water [33,36]. Previous studies suggested that the polycystic kidney disease 1-like 3 (PKD2L1) and PKD1L3/PKD2L1 complexes function as candidate sour taste receptors. However, current evidence indicates that PKD2L1 (TRPP subfamily) is primarily considered a marker of Type III sour-responsive taste cells rather than the proton sensor itself [4,31,33,36]. Additionally, acid-sensing and ion channel subunit 1–3 (ASIC1–3) have been previously proposed to play a role in sour taste stimuli [43]. The expression of ASICs 1a and 1β and PKD2L1 was previously reported on fungiform papillae by immunohistochemistry [43]. Recently, otopeterin-1 (OTOP1) has been described as a proton-selective anion channel engaged in perceiving sour taste modalities [31,33,35,36,44]. This is supported by evidence that the OTOP1 knockout mouse is unable to recognize sour taste; this proton channel is believed to be the main sour taste receptor in humans. OTOP1 is expressed in Type III taste receptor cells and has emerged as a key proton channel involved in sour taste transduction [3,31,33,36]. In the currently proposed model, sour stimuli induce proton entry and intracellular acidification in type III taste receptor cells via OTOP1. Proton-mediated inhibition of inwardly rectifying K^+^ channels (Kir2.1) contributes to membrane depolarization, which activates VGSC and VGCC, increasing intracellular Ca^2+^ levels and triggering neurotransmitter release. Type III cells release serotonin (5-hydroxytryptamine; 5-HT), which mediates communication with gustatory afferent fibers expressing 5-HT3 receptors. In addition to serotonin release, ATP-mediated purinergic signaling may also contribute to sour taste transmission via P2X2/P2X3 on afferent gustatory nerve fibers [31,33,36].

### 3.4. Fatty Taste Receptors

Traditionally, the perception of fat was believed to rely primarily on flavor, along with olfactory and somatosensory cues [45]. However, accumulating evidence suggests that the gustatory system also plays a role in oral fat perception and a proposed taste modality termed oleogustus [7]. A scavenger receptor, CD36, that mediates lipid uptake has been proposed as a candidate for lipid perception in taste bud cells [45]. Upon binding, free fatty acid signaling pathways involving CD36 and the free fatty acid receptor 4 (FFAR4), also known as G-protein-coupled receptor 120 (GPR120), are activated. Further signaling pathways involve α-gustducin and subsequent activation of Ca^2+^-dependent phospholipase (PLC). PLC generates DAG and IP3. DAG can activate the extracellular signal-regulated kinases (ERK) pathway indirectly by phosphorylating protein kinase C (PKC). IP3 binds IP3-gated calcium channels on ER membranes and releases Ca^2+^ from the ER into the cytosol. Increased calcium levels lead to membrane depolarization and activation of TRPM5 channels. Depolarization leads to the release of ATP and, probably, serotonin which serve as neurotransmitters. Both neurotransmitters bind to distinct receptors and transmit the signal for fat perception to the brain via gustatory fibers [46].

**Figure 1:**
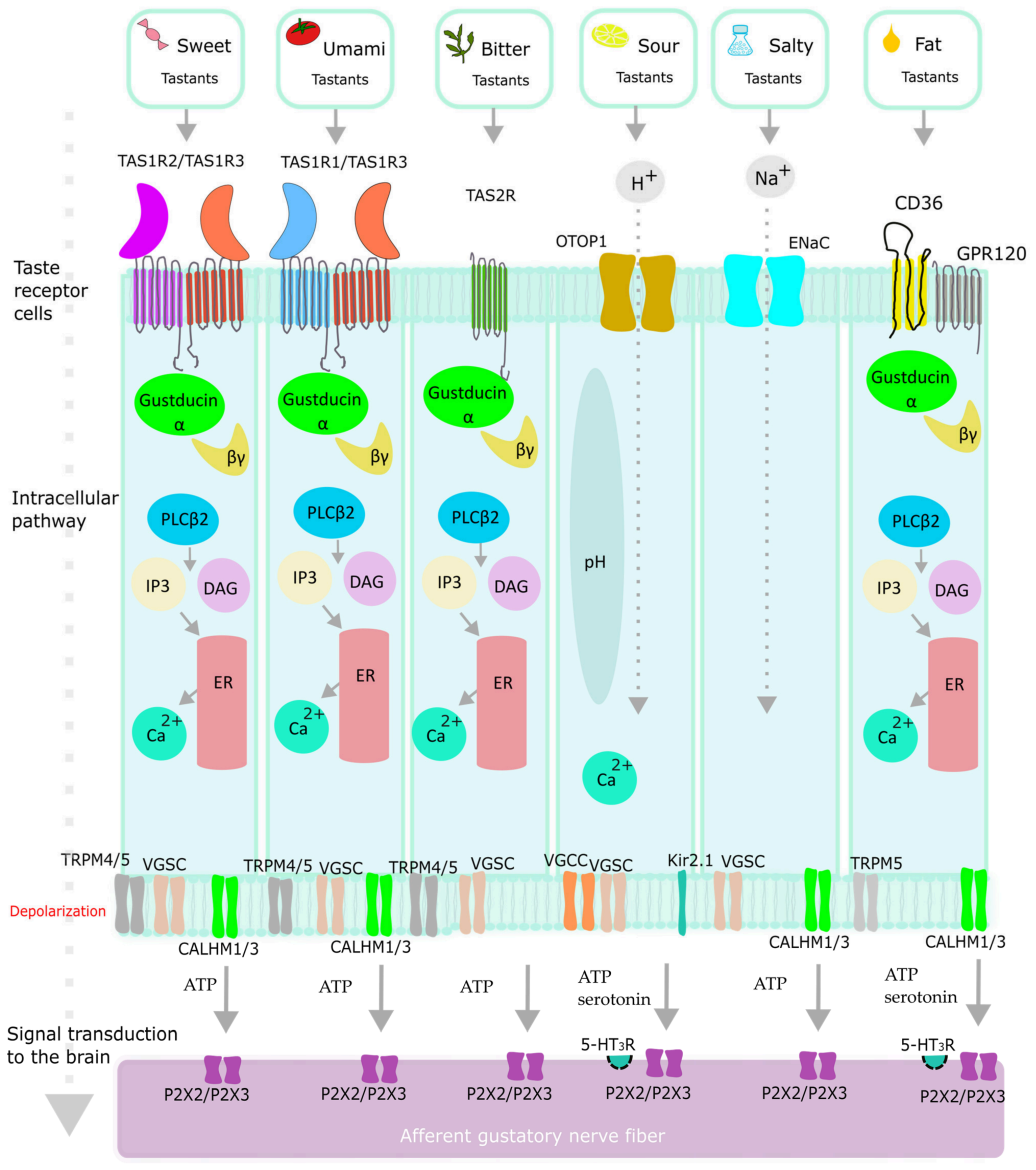
Taste receptors that are engaged in perceiving various taste modalities. Arrows indicate the direction of signaling pathways or ion movement.

Cellular signaling pathways underlying sweet, umami, bitter, salty, sour, and fat taste perception are schematically presented in Figure 1.

### 3.5. Tastants for Taste Receptors

Tastants are soluble molecules that generate a sensory signal by activating specific taste receptors and initiating intracellular signal transduction. The most common tastants associated with each taste modality are given in Table 1.

### 3.6. Expression of Taste Receptors

Taste receptor expression has been found in the oral cavity in taste bud cells, as well as in several extraoral tissues, but mainly at the transcript level. Intriguingly, the genetic variants of these taste receptors might have systemic effects beyond oral taste perception. Data extracted from the Genotype-Tissue Expression Project (GTEx) [54] indicate noticeable extra-oral expression of these receptors across various human tissues, highlighting potentially broader physiological roles (see Figures S1–S3 in the Supplementary Materials). Notably, protein-level evidence for some taste receptors in extraoral tissues remains limited, particularly for TAS receptors, owing to low or inconsistent abundance and challenges in antibody validation.

## 4. Genetic Variants of Taste Receptors Associated with Food-Related Behavior

To date, numerous single-nucleotide variants (SNVs) have been reported in genes encoding various taste receptors. Some of them are related to taste perception or taste preferences, while others also show a similar pattern for particular food liking. Genome-wide association studies (GWAS) have identified multiple genetic loci associated with taste perception and dietary intake, although the number of robust associations for specific taste modalities remains limited [55,56,57]. This may reflect the complex polygenic nature of taste-related traits, as well as the stringent statistical thresholds required for genome-wide significance, which may limit the identification of variants with smaller effects on specific sensory phenotypes. Consequently, GWAS provide important insights into broad genetic architectures, while biologically informed candidate-gene studies may offer complementary information regarding specific molecular mechanisms underlying taste-related traits. Importantly, to prevent overinterpretation, the statistical associations discussed across both candidate and genome-wide frameworks should be viewed with caution; these genetic variations typically represent modest predispositions rather than established causal relationships, and their behavioral expression remains heavily modulated by environmental factors such as age, culture, and clinical status.

### 4.1. Sweet Taste-Related Variants

Dias et al. studied variants of the *TAS1R2* gene in relation to sweet taste perception and sugar intake. Authors genotyped rs12033832, rs12137730, rs35874116, rs3935570, rs4920564, rs4920566, rs7513755 and rs9701796. The rs12033832 (G/A) was associated with both sucrose taste perception and sugar intake. The observed association was body mass index (BMI)-dependent, and G allele carrier individuals with a BMI ≥ 25 exhibited lower sucrose sensitivity, higher sucrose thresholds, and higher sugar intake. In contrast, the direction of the association differed in individuals with a BMI < 25. In this study, rs3935570 also affected taste but not sugar intake [58]. Eny et al. performed a study on non-diabetic and diabetic individuals to determine the relationship between *TAS1R2* genotypes and dietary intake. Consumption of sugar in the non-diabetic population was associated with the Ile191Val (rs35874116) *TAS1R2* gene polymorphism, but the effect of genotype was observed only in individuals with a BMI ≥ 25 [59]. Compared with Ile allele homozygotes for rs35874116, Val allele-bearing individuals consumed less sugar in both studied groups [59]. Interestingly, a recent study by Serrano et al. suggested that the rs35874116 variant may positively influence muscle function and metabolism [60]. In the study by Han et al., individuals with the GG genotype at rs12033832 in *TAS1R2* consumed more carbohydrates than AA homozygotes. Carriers of the C allele at the rs38574116 SNV exhibit higher intake of sweet food as compared to the TT genotype. *TAS1R3* polymorphism was not associated with sweet taste and sweet food intake [61]. A study by Fushan et al. demonstrated that taste sensitivity to sucrose correlates with two SNVs, rs307355 and rs35744813, which lie upstream of the *TAS1R3* coding sequence [62]. Ramoz-Lopez et al. conducted a cross-sectional study of the West Mexican population to analyze the *TAS1R2* (Ile191Val) rs35874116 polymorphism and food intake. Authors documented that carriers of the Val/Val genotype exhibit higher intake of total carbohydrates, as well as of cereals and vegetables, than carriers of other genotypes. Alongside Val/Val carriers exhibit a higher risk for hypertriglyceridemia [63]. In the study by Pawellek et al., the authors genotyped the *TAS2R38* first PAV haplotype (further discussed in Section 4.3) site (rs713598) in a population of 1 to 6-year-old children from various European countries. They confirmed that this genetic variant was related to the intake of sweet-tasting foods. Carriers of PP and PA genotypes consumed a higher amount of sweet food compared to AA homozygotes and consumed more energy-dense, sweet-tasting food [64]. In a study of taste preferences in the Lithuanian population, Kavaliauskiene et al. confirmed three variants of the genes *TAS1R3* (rs35424002), *TAS1R2* (rs9988418), and *GNAT3* (rs10230573) as related to sweet taste preferences [65]. Interestingly, Rawal et al. suggested in the study a broader taste perception for TAS1R1. Authors revealed that carriers of two SNVs, an intronic rs17492553 and an exonic rs34160967 SNV in *TAS1R1*, perceive prototypical tastants, e.g., sucrose, NaCl, citric acid, and quinine hydrochloride with modest intensity [66]. In a study by Joseph et al., children carrying the A allele of rs1726866 (V262A) and/or the V allele of rs10246939 (I296V) were more sensitive to sweet taste perception [67].

Common variants related to sweet taste perception are summarized in Table 2.

### 4.2. Umami Taste-Related Variants

Shigemura et al. investigated the relationship between recognition thresholds for umami tastes, e.g., L-glutamate and monosodium glutamate (MSG), and genetic variants in *TAS1R1* (rs34160967) and *TAS1R3* (rs307377) which form the umami receptor, and demonstrated that umami taste perception is associated with specific genetic variants of these genes [68]. A study of Italian subjects demonstrated that the *CD36* rs1761667 A-allele, previously associated with fat perception, was also associated with decreased liking for umami foods. In contrast, UK subjects in the second study demonstrated increased umami taste perception in CD36 rs1761667 A-allele carriers [69]. Raliou et al., in a study of human umami taste perception, demonstrated that variants C329T (rs41278020) in *TAS1R1* and C2269T (rs35744813) in *TAS1R3* were associated with nontaster status. At the same time, the G1114A (rs34160967) SNV in *TAS1R1* was associated with umami taster status [70]. Recently, Neves et al. investigated taste preferences and dietary characteristics in a group of infants undergoing complementary feeding [71]. Authors demonstrated that a preference for umami-tasting food was associated with carriers of the rs846672 SNV in the *TAS2R16* gene [71]. Chen et al. studied umami taste perception with monopotassium glutamate (MPG) and observed that carriers of the rare T allele R757C (C/T, rs35744813) in the *TAS1R3* gene perceived MPG with higher intensity. Umami perception, estimated by higher solution concentration of MPG, was characteristic for carriers of the A allele of A5T (rs307355) and the A allele of R247H (rs34160967) for *TAS1R3* genes [72].

Common variants related to umami taste perception are given in Table 3.

### 4.3. Bitter Taste-Related Variants

Kim et al. in 2003 mapped the ability to perceive a bitter tastant called phenylthiocarbamide (PTC) to the *TAS2R38* region of the gene [73]. Authors identified three SNVs that explained the distribution of PTC taste intensity. Two predominant haplotypes were defined, A49P (Alanine/Proline; AP) rs713598, V262A (Valine/Alanine; VA) rs1726866, and I296V (Isoleucine/Valine; IV) rs10246939. The nontaster haplotype AVI and the taster haplotype PAV are reported to account for about 50% of each haplotype in the European population [73]. It has been observed that supertasters for bitter taste have more fungiform papillae, are also more sensitive to sweet tastes, and oral burn derived from intake of oral irritants and oral tactile perception of viscosity [74,75]. Fisher et al. in their experiment substituted PROP for PTC to avoid the sulfurous odor sensed by PTC [75]. PROP bitter sensitivity resembles that of PTC. However, according to Hayes et al., additional bitter receptors may be engaged in PROP bitterness [76]. Haydar et al. conducted a GWAS of liking various modalities, including the bitter taste of rocket salad. They observed that homozygotes with the PAV/PAV haplotype (rs10246939, rs1726866, and rs713598) like rocket salad less than individuals with the AVI/AVI haplotype [56]. In the Neeves study, bitter taste reactions were connected with *TAS1R2* (rs9701796) and *TAS1R3* (rs307355) polymorphisms [71]. Risso et al. studied taste perception, taste, and food preferences in four diverse populations, including Italy, the Maghreb, Northern Europe, and Sri Lanka [24]. Authors demonstrated that rs10246939, rs1726866, and rs713598 of *TAS2R38* were associated with broccoli score in the studied groups. An inverse correlation was documented between the PAV haplotype and the reported broccoli score. The PAV taster haplotype also associates with PROP bitterness. Individuals who could perceive the bitter taste of stevioside were more frequently carriers of the G allele at rs2234001 in *TAS2R4*. *TAS2R16* rs860170 SNV A allele was associated with perceiving the bitterness of salicin. The analyzed population showed different food habit scores for broccoli, mustard, beer, licorice, and Parmesan cheese [24]. Phytochemicals known as glucosinolates are naturally occurring sulfur-containing compounds from the *Brassicaceae* family (e.g., broccoli, cabbage, cauliflower, etc.). When plant cells are damaged by chewing or chopping, the endogenous enzyme myrosinase hydrolyzes glucosinolates and produces isothiocyanates. Some individuals sense isothiocyanates as bitter and pungent and avoid food containing these compounds [77]. Drewnowski et al. distinguished PROP nontasters, tasters, and supertasters according to detection thresholds and intensity ratings in a population of young women [23]. Authors demonstrated that subjects with greater PROP sensitivity showed less acceptance of cruciferous vegetables, tart citrus fruits (grapefruits, rhubarb, lemons), coffee, and whole-grain bread. Supertasters for PROP also showed lower acceptance of soft fruits and berries than PROP nontasters [23]. In the other study, the authors focused on the remaining receptors in the TAS2R family to explore the roles of their genetic variants and preferences for beverages [78]. Carriers of the AA genotype of SNV C/A rs846672 in the *TAS2R16* gene revealed the association with the frequency of consuming alcoholic beverages and the amount of alcohol intake. Regarding carriers of C/G SNV rs1308724 in *TAS2R16*, CC homozygotes for this polymorphism tend to consume alcohol less frequently than other genotypes [78]. Duffy et al. performed a study linking *TAS2R38* PAV/AVI haplotypes and better perception of PROP with alcohol use. Authors found that the higher alcohol intake was associated with individuals carrying AVI/AVI homozygous genotypes compared to PAV/AVI heterozygotes or PAV/PAV homozygotes. According to the authors, after controlling for age, more variance in alcohol use was explained by bitter PROP tasting than by the *TAS2R38* genotype [38]. Hayes et al. identified four SNVs (rs2234001, rs2227264, rs765007, and rs2234012) in the *TAS2R3*, *TAS2R4*, and *TAS2R5* genes, which are responsible for coffee bitterness, and 89% of the studied samples showed three common haplotypes. It was found that individuals with the more responsive haplotype TGAG perceive bitterness twice as much as those with the less responsive haplotype CCGT [78]. Grapefruit juice bitterness in this study was associated with the rs10772420 A/G in *TAS2R19*, resulting in the substitution of (Cys299Arg). Cys299 homozygotes reported grapefruit juice to be bitterer than Arg299 carriers [78]. According to Roudnitzky et al., in the perception of bitter aftertaste of sweeteners, both saccharin and aspartame K contribute more to the *TAS2R31* than to the *TAS2R43* locus [50]. Pirastu et al. suggested that TAS2R43 is involved in coffee liking and found a relationship between caffeine perception and the H212R (rs71443637) variant of *TAS2R43* [79]. In the study by Allen et al., rs10772423 in *TAS2R31* and rs3741845 in *TAS2R9* were associated with the bitterness of acesulfame K [80].

Common variants related to bitter taste perception are listed in Table 4.

### 4.4. Salty Taste Related-Variants

Salty taste in the context of gene variants was studied by Dias et al. in genes encoding ENaC channel subunits. The authors found evidence for two SNVs in the *SCNN1B* gene that modified salt taste perception. In this study, carriers of the AA genotype for SNV rs239345 (A/T) and carriers of the T allele of rs3785368 (C/T) reported perceiving salt solutions less intensely than carriers of other alleles. Moreover, SNV in the *TRPV1* gene was also suggested as a candidate to modify salt taste perception [81]. Another study conducted by Mohammadiffard et al. in the Iranian population found higher salt intake in carriers of the SNV rs239345 A allele compared to TT homozygotes in the *SCNN1B* gene. In this study, carriers of the TT genotype of *TRPV1* (rs224534) consumed less sodium compared to the CC genotype [82]. In the study by Tapanee et al., carriers of the AA genotype at SNV rs4790522 of *TRPV*1 with hypertension and obesity showed lower salt taste sensitivity [83]. Importantly, variants within the *TAS1R1* also exhibit multimodal characteristics affecting the perception of multiple prototypical stimuli (including NaCl and quinine hydrochloride) [66]. To avoid narrative redundancy, these specific polymorphisms were already noted and contextualized in the preceding section on sweet taste modalities (see the Section 4.1). A study by Deshawere et al. reported an association between *TAS2R38* PAV/AVI haplotypes and perceived salt intensity, with PAV/PAV individuals rating NaCl solutions as more intense than PAV/AVI and AVI/AVI carriers [84].

Common variants related to salty taste perception are listed in Table 5.

### 4.5. Sour Taste-Related Variants

To our knowledge, there is limited data on *OTOP1* variants and their relationship to food-related behavior. Regarding oral cavity health status, De Jesus et al. recently documented an association between allelic variants rs145781170 and rs17697262 in the *OTOP1* gene and severe dental caries in children [85]. The former polymorphism was also associated with the oral fungal genus *Blumeria* [85]. Interestingly, in a study of infants undergoing complementary feeding, sour food preferences were associated with a variant in the *TAS1R3* gene (rs35744813) [71].

### 4.6. Fatty Taste-Related Variants

Pepino et al. studied the rs1761667 SNP in the *CD36* gene in relation to oleic acid (FA) and triolein (TGA) detection thresholds. Homozygotes for the GG rs1761667 SNV showed lower detection thresholds than AA homozygotes, accompanied by lower CD36 expression [45]. There were no differences in the fat preference scores estimated in this study. Melis et al. conducted a study of nontasters and supertasters to examine PROP responsiveness and found that both PROP responsiveness and fatty acid perception were associated with the rs1761667 SNP in *CD36* [86]. Specifically, the authors demonstrated in their study that GG homozygotes for rs1761667 showed a greater sensitivity to oleic acid than AA homozygotes [86]. In the study by Mrezik et al., obese Tunisian women carrying the GG genotype at rs1761667 had a lower detection threshold for oleic acid than AA homozygotes [87]. Sayed et al. conducted a study on obese and lean Algerian children. They observed that, among obese children who were carriers of the *CD36* A allele, the lipid taste perception threshold was higher than among G-allele carriers [53]. Keller et al. conducted a study among African-American individuals. They found that AA genotype carriers of rs1761667, as well as CT or TT genotype carriers of rs1527483, perceived more fat content in salad dressings. The former group also revealed a higher mean acceptance of added fats and oils than other genotypes [88]. In another study, individuals with the rs1761667 SNV in *CD36* showed similar fat preferences and fat-food cravings [45]. Ong et al., in a study of Malaysian individuals, demonstrated that individuals carrying the rs1527483 TT genotype perceived custard as creamier and cream crackers as higher in fat content. Also, individuals with the rs1527483 TT genotype and the T allele reported significantly greater fat content in cream crackers [89]. In the study by Piluso et al., rs1761667 A allele carriers showed a lower liking for the fatty food group, namely cheeses. However, the authors found no association between this polymorphism and the intensity of fat perception [69]. In the study by Muthuswamy at al. participants with minor alleles of *CD36* rs1527483 and rs1761667 consumed higher amounts of saturated fat [90].

Common variants related to fatty taste perception are given in Table 6.

**Figure 2:**
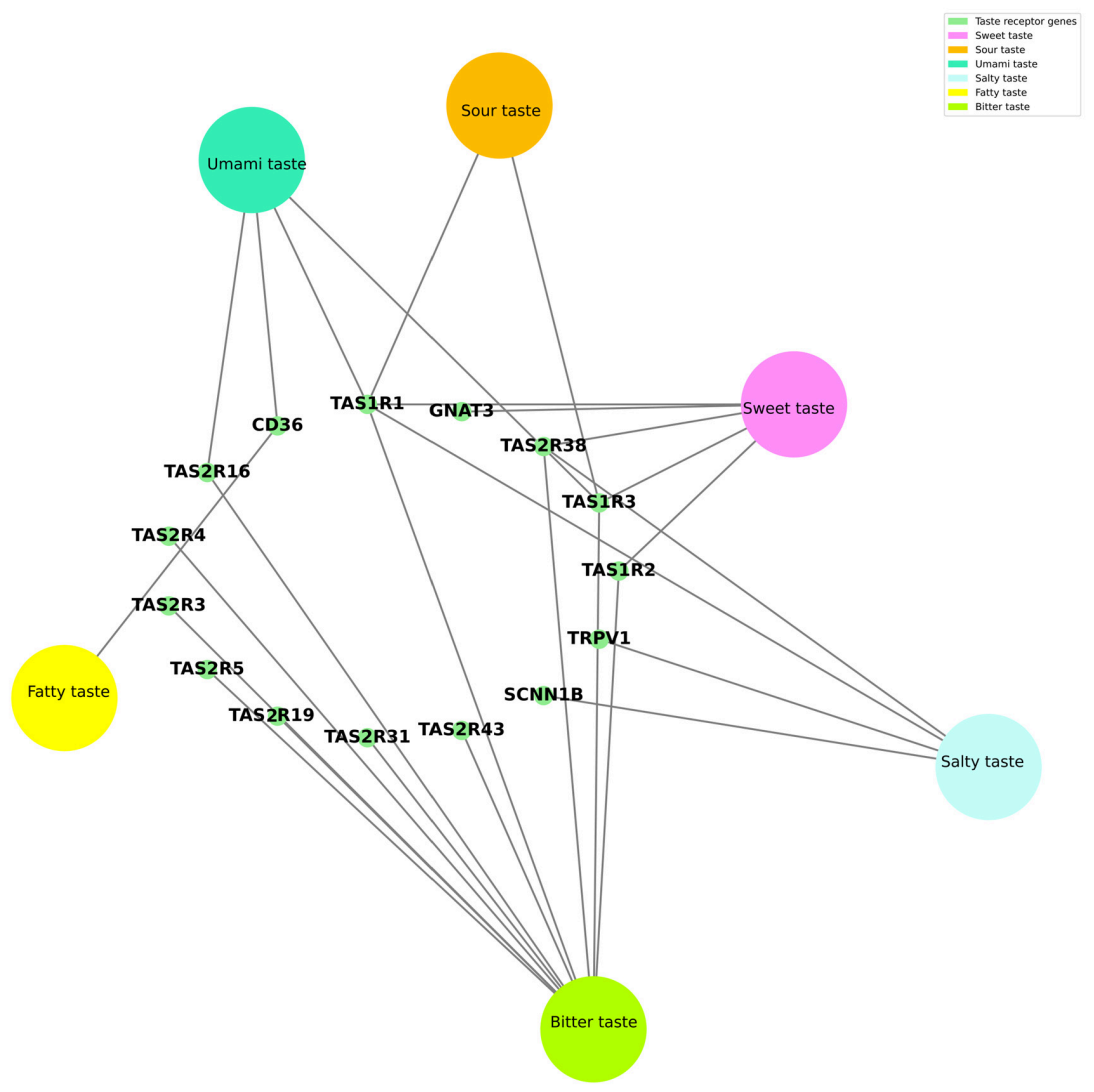
Radial network visualization of taste receptor genes and associated taste modalities. The network graph illustrates the relationships between taste receptor genes (smaller green nodes) and particular taste modalities (colored nodes). Taste modalities nodes are arranged on the outer circle, while associated taste receptor gene nodes are positioned radially around each taste modality to visualize direct connections. Edges between genes and taste modalities represent observed associations. Data were visualized using NetworkX 3.5 Python 3.12. package.

Figure 2 provides a schematic summary of taste receptor genes and their associations with taste modalities.

## 5. Non-Taste Modulators of Food-Related Behavior

In addition to taste receptor genes, genetic variation in metabolic and reward-related pathways has been proposed to modulate food-related behavior [18]. These effects are considered indirect, acting through mechanisms related to energy balance regulation, reward processing, and hedonic responses rather than primary taste perception. Recent large-scale GWAS of food liking have revealed a hierarchical genetic structure of dietary preferences, suggesting a partial dissociation between sensory-related traits and reward-driven or learned food preferences [55]. Furthermore, many genetic loci involved in food-related behavior exhibit pleiotropic effects, influencing not only dietary preferences but also broader metabolic and neurobehavioral traits. In particular, non-taste pathways have been implicated in cardiometabolic phenotypes and reward-related processes, which are also relevant to addiction-related behaviors, highlighting the complex genetic architecture underlying food-related behavior [55,91,92,93]. Importantly, the statistical associations derived across these chapters—spanning both targeted candidate-gene platforms and selective genome-wide screens—should be understood as contributing to subtle behavioral predispositions rather than establishing direct causal links, as their practical phenotypic expression remains heavily intertwined with lifestyle, environmental exposures, and metabolic health status.

### 5.1. Genes Involved in Metabolic Regulation

#### 5.1.1. *FTO*

The fat mass and obesity-associated protein (FTO) is an RNA m6-methyladenosine (m6A) demethylase. FTO is expressed in a variety of tissues, with predominant expression in the hypothalamic nuclei. GWAS have consistently linked polymorphism in FTO with BMI and obesity [94]. In a study of the Spanish population, the authors found that TT genotype carriers of the *FTO* rs9939609 polymorphism showed higher post-exercise appetite and hunger than AT genotype carriers [95]. Harbron et al. demonstrated that carriers of the *FTO* rs17817449 GG genotype consumed more refined starches and high-fat foods [96]. Madrigal-Juarez et al. conducted a study on the Western Mexico population. They demonstrated that individuals with the AA and AT rs9939609 genotypes consumed more added sugar than those with the TT genotype [97]. In a 2-year randomized clinical trial involving overweight participants, Huang et al. observed that individuals with high protein intake and the A allele of *FTO* rs9939609 had lower food cravings and lower appetite scores. There was no such association in the case of the subject on a low-protein diet [98]. In a genome-wide association meta-analysis, Tanaka et al. demonstrated that the risk *FTO* variant (rs1421085) was associated with higher protein intake [57]. As reported by Chuang et al. CC homozygotes of rs1421085, an obesity associated C allele, exhibit increased fat intake over time during 70–80 years of age while aging [99]. In a large GWAS, rs10163409 in *FTO* was associated with the percentage of total caloric intake from protein and carbohydrates [100].

#### 5.1.2. *MC4R*

Melanocortin 4 receptor (MC4R) is a member of the GPCR superfamily. MC4R is mainly expressed in the central nervous system, including the hypothalamic arcuate nucleus and paraventricular nucleus, as well as in extra-brain tissues. MC4R plays a significant role in regulating food intake, appetite, and energy metabolism. Mutations in this gene lead to severe obesity [101]. Klaauw et al. conducted a study evaluating food preferences and taste preferences in lean and obese individuals bearing *MC4R* mutations. Subjects with MC4R deficiency preferred a higher-fat meal than lean, and obese controls without *MC4R* mutations. Regarding sweet taste preferences, subjects with *MC4R* variants consumed fewer sucrose meals and showed lower liking ratings for high-sucrose meals than lean and obese control subjects [102]. Huang et al. in a 2-year POUNDS Lost (Preventing Overweight Using Novel Dietary Strategies) trial genotyped *MC4R* rs7227255 SNV to estimate its relationships with appetite behavior. Authors found that subjects carrying the A allele of this polymorphism showed increased appetite and craving behavior in the high-protein weight-loss diet group [103]. In GWAS, variants in *MC4R* were also associated with coffee intake, with the rs66723169 A allele also liking coffee without sugar and disliking tea with sugar added [104].

#### 5.1.3. *GHRL*

Ghrelin is a peptide hormone predominantly produced by enteroendocrine X/A-like cells of the stomach, with lower levels of expression in several extra-gastrointestinal tissues, including the arcuate and paraventricular nuclei of the pituitary, the lung, kidney, and bone. Ghrelin is involved in the regulation of food reward-related behaviors and carbohydrate and lipid metabolism [105,106,107]. The study by Santos et al. in obese individuals who underwent a nutritional program aimed to assess the impact of various genetic variants on food intake and demonstrated that the *GHRL* rs26311 G allele was associated with higher post-program protein intake compared with the CC genotype [108]. Takezawa et al. analyzed dietary behavior in a group of obese Japanese women. They documented that subjects carrying the CC genotype of the 3056T/C SNV (rs2075356) exhibit lower sugar, dairy, and total food consumption. Also, homozygotes CC for −1062G/C (rs26311) were light eaters despite being predisposed to obesity [109].

#### 5.1.4. *FGF21*

Fibroblast growth factor 21 (FGF21) is chiefly produced in the liver endocrine hormone that impacts regulation of metabolism targeting several tissues including adipose tissue, pancreas, and central nervous system. Holstein-Rathlou et al. suggested that FGF21 modulates sugar and alcohol intake by acting through a negative feedback loop between the liver and the brain [110]. Soberg et al. conducted a study in a large Danish cohort and found that a *FGF21* polymorphism is associated with sweet preferences. In this study, individuals carrying the A allele of rs838133, a *FGF21* SNV, showed increased preference for sweet-tasting food [111]. Janzi et al., in a large Swedish cohort, found that rs838145, rs838133, and rs8103840 SNVs in *the FGF21* locus are high linkage disequilibrium (LD). G, A, and C alleles, respectively, were related to consuming total sugar, added sugar, and sweet taste sugars, including sucrose and all monosaccharides [112]. In a large GWAS conducted by Chu et al., the rs838133 SNV in *FGF21* and the rs10163409 SNV in *FTO* were significantly associated with the percentage of total caloric intake from protein and carbohydrate [100].

#### 5.1.5. *ADRB2*

*ADRB2* encodes the beta2 adrenergic receptor, which is widely expressed across tissues. ADRB2 is involved in smooth muscle relaxation and metabolic processes, including glycogenolysis and lipolysis of adipose tissue [113,114]. In a self-reported questionnaire study conducted by Narita et al. in Japanese adults, Gly16Arg (G/C) genotypes of *ADRB2* were associated with a preference for sour foods, e.g., grapefruit and pickled Japanese plum, in male subjects [115]. Significant differences were also observed among *ADRB2* genotypes for pickled vegetables, grapefruit, and coffee in the female population [115].

### 5.2. Genes Involved in Reward-Related Pathway

#### 5.2.1. *DRD2*

The dopamine D2 receptor, encoded by the *DRD2* gene, is a member of the GPCR family. DRD2 is widely expressed in the central nervous system, especially in the striatum and other reward-related brain regions. DRD2 plays a central role in dopaminergic signaling, regulating motivation, reward processing, and inhibitory control of behavior [116]. The *ANKK1*/*DRD2* locus includes the *DRD2* receptor and adjacent gene ankyrin repeat and kinase containing domain 1 (*ANKK1*), which may influence dopaminergic signaling and has been associated with reward-related eating behavior and food preferences [116,117]. In the study conducted by Antonova and Spasova, the *ANKK1*/*DRD2* rs1800497 SNV, Taq 1A (C/T), was used to evaluate genetic predisposition to appetite behavior [118]. The other study conducted on Indian Malaysian and Chinese university students involved estimation of eating behaviors and genotyping of *ANKK1*/*DRD2* rs1800497 SNV, Taq 1A, (C/T), intronic SNV Taq1B G/A rs1079597 and intronic SNV Taq1D C/T; also known as rs1800498 G/A. Significant differences were found among Taq1 genotypes in food preferences. Individuals with alleles A1/A1 and B1/B1 showed a stronger preference for fast food. Individuals with the D1 allele showed higher starchy food craving and a preference for “mamak style” food [119]. Obregon et al. studied eating behavior in association with Taq1 polymorphism in Chilean normal-weight, overweight, and obese adults. Authors found that women who were carriers of the A1 allele of the Taq1 SNV showed higher scores for emotional eating and snack food reinforcement than women with other genotypes [120]. In the study of Jabłoński et al., in men with alcohol dependence, particular alleles of Taq1A SNV were associated with sucrose preferences. A2 alleles were more frequent in sweet dislikers, while A1 alleles were more prevalent in sweet likers [121].

#### 5.2.2. *OPRM1*

The mu opioid receptor (MOR) encoded by the gene (*OPRM1*) belongs to the GPCR family and is widely expressed in the central nervous system, including brain regions involved in reward processing. It plays a key role in modulating pain perception, stress response, and reward-related behaviors, particularly those linked to food intake and hedonic eating [122,123]. Chmurzyńska et al. found no association between high-fat food intake and the *OPRM1* variant rs1799971. However, authors noted an association between the minor allele G of this variant and lower fast-food intake in individuals with high hedonic hunger [123]. In the study by Davis et al., haplotypes containing rs495491 and rs563649 were associated with food preferences. In this study, stronger sweet and fatty food preferences were observed in individuals with the A–C haplotype compared to those with other haplotypes [122].

### 5.3. Other Genes

#### 5.3.1. *CYP1A2*/*AHR*

Cytochrome P450 family 1 subfamily A member 2 (*CYP1A2*) encodes a hepatic cytochrome P450 monooxygenase that is the primary hepatic enzyme responsible for approximately 95% of caffeine metabolism [104,124]. Coffee, tea, cocoa, and kola nuts constitute the major natural dietary sources of caffeine. The genetic associations observed for both coffee and tea consumption are largely driven by caffeine metabolism, as caffeine is the principal bioactive compound shared by both beverages. *CYP1A2* genetic variation contributes to slow and rapid caffeine metabolizer phenotypes, reflecting differences in enzyme activity and caffeine clearance. Rapid metabolizers eliminate caffeine more efficiently, whereas slow metabolizers maintain higher circulating caffeine concentrations for longer periods. These metabolic differences may influence coffee and tea consumption patterns, as individuals with faster caffeine clearance may consume greater amounts to achieve the desired stimulant effect. The expression of the *CYP1A2* gene is regulated by the aryl hydrocarbon receptor (AHR) pathway. AHR functions as a transcriptional regulator of xenobiotic-metabolizing enzymes, including CYP1A2, through binding of the AHR–AHR nuclear translocator (ARNT) AHR–ARNT complex to xenobiotic response elements (XREs). Consequently, variants in genes involved in caffeine metabolism, particularly *AHR* and *CYP1A2*, have been consistently identified as established loci for both coffee and tea preferences [124]. Cornelis et al. conducted a meta-analysis of GWAS summary statistics from various ancestral populations to identify variants associated with coffee consumption. In this study, rs2472297 and rs2470893 SNVs in the *CYP1A1*/*CYP1A2* locus, and rs4410790 and rs6968554 in *AHR*, were associated with the number of cups of coffee consumed per day [125]. Genetic variants located near *AHR* (rs4410790) and *CYP1A2* (rs2472297) have been linked to increased tea consumption, with the C allele associated with higher intake [104]. A meta-analysis of four GWAS on coffee intake across various populations by Sulem et al. identified rs2472297 in the *CYP1A1*/*CYP1A2* locus and rs6968865 in *the AHR* locus as polymorphisms associated with coffee consumption [126]. Other studies confirmed that *AHR* and *CYP1A2*/*CYP1A1* loci are associated with habitual caffeine consumption [124,127,128]. Notably, Furukawa et al. conducted a GWAS of habitual black tea consumption in the Japanese population. They identified that gene variants at the 12q24 locus were associated with black tea consumption in Japanese populations [129].

#### 5.3.2. *SLC4A5*

*SLC4A5* encodes the electrogenic sodium-bicarbonate cotransporter NBcE2, a membrane transporter involved in bicarbonate transport and pH regulation. This contrasporter is linked to salt-sensitive hypertension [130], in the study conducted by Pilic et al. SNV in *SLC4A5*, namely rs10177833 C/A, was found to be related to salt intake. Authors reported that the trend towards increased energy-adjusted Na intake was associated with the increasing number of A alleles in carriers of this polymorphism [131].

#### 5.3.3. *CA6*

A secreted salivary enzyme, carbonic anhydrase VI (C6), called gustin, contributes to buffering capacity in the oral cavity. In this way, CA6 may indirectly influence taste perception by modulating the chemical environment surrounding taste receptor cells [132]. Several studies confirm the association of *CA6* genetic variants with taste perception, taste preferences, and food preferences. Perceiving salt tastants was associated with, in the study by Feeney et al. [133]. Authors documented that the Asn256 SNV rs3737665 and the Gly287Glu SNV (rs3765964) in the *CA6* gene were associated with differences in the perceived intensity of NaCl [133]. Responsiveness to PROP was inversely related to salivary zinc ion concentrations and directly associated with polymorphism rs2274333 (A/G). Padiglia et al. studied PROP perception and found an association between PROP taste perception and rs2274333 (A/G) of *CA6*. Supertasters with the highest PROP responsiveness were carriers of the AA genotype for this variant [134]. Bell et al. studied the perception of rucola (*Eruca sativa*) flavor. In this study, individuals possessing at least one *TAS2R38* PAV allele perceive fewer flavors than subjects with the AVI/AVI genotype. Individuals possessing supertaster PAV alleles along with the rs2274333 *CA6* A allele demonstrated less potential to perceive flavors and aromas of *Eruca* leaves due to stronger perception of bitter taste [77]. Notably, rs2274333 was found to influence fungiform papilla features in a study by Melis et al. [135] The authors showed, *in vitro*, that cells treated with saliva from individuals with the AA genotype of the gustin gene showed increased proliferation and metabolic activity compared to cells treated with saliva from individuals with the GG genotype. Moreover, cells treated with the active isoform of gustin showed increased metabolic activity, whereas those treated with the inactive isoform showed decreased metabolic activity. Authors documented that the gustin genotype was also associated with papilla diameter and found that individuals with the GG genotype had papillae with greater shape variation [135].

#### 5.3.4. *AMY1*

Saliva is rich in proteins, the most abundant of which is amylase I. Amylase-1 (AMY1) is an enzyme that is responsible for the initial digestion of starch in the human cavity. The copy number variants (CNV) of *AMY1* are related to both the concentration and activity of this enzyme. Differences in CNV of *AMY1* among individuals may contribute to initial sweet taste perception after starch digestion and, subsequently, to dietary starch intake [136,137]. Mandel et al. demonstrated a significant correlation between salivary amylase amount in ml and *AMY1* gene copy number. An increase in gene copy number was accompanied by higher salivary amylase activity. Authors also evaluated the relationship between *AMY1* gene copy number and perceived starch viscosity. However, the number of gene copies was not associated with either the overall change in perceived viscosity over time or the time to reach half perceived starch viscosity [136].

#### 5.3.5. *RETN*

Resistin is a peptide encoded by the *RETN* gene known as an adipokine, but mainly expressed in macrophages [138]. Resistin is believed to act as a proinflammatory factor in metabolic processes [139]. Recently, our studies have shown that the resistin SNV (−420G/C; rs1862513) is associated with endothelial dysfunction and salt taste preference (liking) in hypertensive patients [139]. In addition, Allele G and the CG genotype of the resistin SNV (−420C/G) were linked to fried-food taste preferences in patients with hypertriglyceridemia [140]. A recent study by Santos et al. documented that carriers of the *RETN* rs3745367 A allele exhibit increased basal intake of protein, monounsaturated fatty acids (MUFAs), saturated fatty acids (SFAs), and cholesterol compared with subjects with the GG genotype [108].

#### 5.3.6. *MCM6*/*LCT*

*LCT* encodes the enzyme lactase-phlorizin hydrolase. This enzyme digests lactose; however, its activity varies across populations worldwide [141]. It leads to lactase persistence when lactose is digested in adulthood, or to lactase non-persistence when lactose and dairy are less well tolerated. The minichromosome maintenance complex component 6 (*MCM6*) gene is located near the *LCT* gene and contains regulatory enhancer elements that control *LCT* expression [39,142]. The lactase persistence variant at the *MCM6* locus, rs4988235 (C/T), CC carriers tolerate lactose much less than the remaining genotypes [143]. Notably, the study by Qin et al. analyzed four bacterial taxa strongly associated with the *LCT* locus in subjects carrying rs4988235 genotypes in the context of dairy diets [144]. Authors reported a significant increase in *Bifidobacterium* abundance in CC genotype carriers who reported a regular dairy diet, suggesting that the microbiota is an additional factor influencing host genetic predisposition to food preferences [144]. Outstandingly, Pirastu et al. performed a GWAS on liking of four categories of foods, among them dairy foods, and identified additional, different variants linked to other genetic regions located on chromosomes 2, 5, and 22 that were associated with liking of particular foods such as blue cheese (rs12994253), ice cream liking (rs2035613), and plain yogurt liking (rs4239891) [145].

#### 5.3.7. *IZUMO1*

Tanaka et al. performed a GWAS meta-analysis of observational studies and found that the *IZUMO1* locus was among those associated with fat consumption [57]. The gene encodes a sperm-specific membrane protein essential during fertilization. Thus, the biological mechanism underlying this association remained unclear. Notably, *IZUMO1* is located in proximity to the *FGF21* gene [39]. The proximity of I*ZUMO1* to *FGF21* is noteworthy because FGF21 is involved in metabolic regulation and food-related behavior, including sugar preference. However, the contribution of *FGF21* or other elements within this locus to the observed association remains unclear.

**Figure 3:**
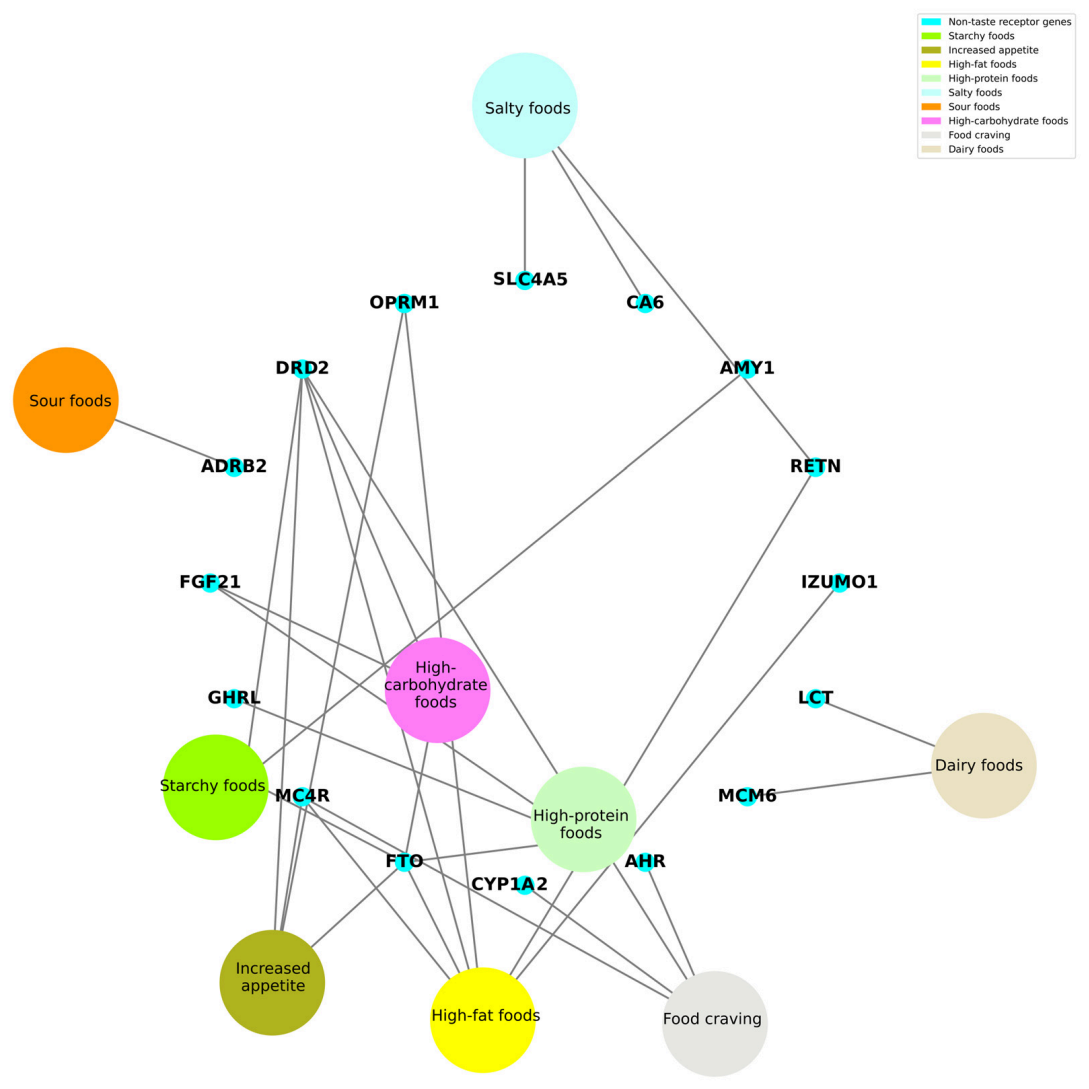
Radial network visualization of non-taste receptor genes and associated food-related traits. Food-related traits were grouped into broader categories for visualization purposes. The network graph illustrates the relationships between non-taste receptor genes (smaller blue nodes) and food-related traits (colored nodes). Food group nodes are arranged on the outer circle, while associated non-taste receptor gene nodes are positioned radially around each food group to visualize direct connections. Edges between genes and food groups represent observed associations. Data were visualized using NetworkX 3.5, Python 3.12. package.

Figure 3 summarizes schematically non-taste receptor genes and their associations with food-related traits.

## 6. Crosstalk Between Health Status, Genetic Variants, and Food Related Behavior

Alteration of taste perception may be associated with a wide range of conditions, including metabolic, neurological, infectious, autoimmune, and medication-related disorders [19,77,146,147,148]. This may subsequently lead to a change in food-related behavior. More than half of patients suffering from chronic kidney disease (CKD) report impaired salt sensitivity. Fewer taste buds, altered saliva composition, including urea and zinc deficiency, may lie upon the mechanism of taste perception in CKD [148]. In the GWAS performed by Fernandez et al., taste perception, taste preferences, and food preferences were analyzed in this population [146]. Individuals with type 2 diabetes mellitus (DM2) showed lower preferences for all five tastes, as measured by the combined total taste score, and lower bitter taste perception. Regarding taste preferences, the study found a higher preference for sweet taste than in non-diabetic subjects [146]. Moreover, authors noticed a strong relationship between sweet taste preferences and liking sweet-tasting foods in DM2 individuals [146]. Regarding obesity, the influence of taste and food preferences has been observed, including reduced taste sensitivity and a preference for fat. However, findings regarding sweet taste preferences remain inconsistent across studies [21,149]. Interindividual differences in sweet-liking phenotypes, age, exposure to obesogenic environments, alterations in sensory-specific satiety, and methodological differences among studies may explain the inconsistent findings regarding sweet preference in obesity [21,149]. During viral COVID-19 infection, taste and olfactory alterations also have been reported. Taste disturbances are prevalent in COVID-19. They may be primarily driven by olfactory dysfunction, leading to impaired flavor perception; thus, COVID-19 is also associated with chemosensory impairment [20,150]. In addition to pathological conditions, physiological states may also contribute to changes in taste perception and taste-related behavior. During the menstrual cycle in females, during the luteal phase, women tend to intake more energy than in the phase preceding ovulation. This phenomenon is likely to be mediated by progesterone and estrogen levels and their influence on appetite behavior [151,152]. Alberti-Fidanza et al. conducted a study of gustatory and food-habit alterations during the menstrual cycle and measured sex hormone levels in 8 women. Elevated estradiol levels were associated with increased sensitivity to sweet taste. An elevated progesterone level was associated with increased bitter taste. Alterations in food habits during the menstrual cycle involved meat and fruit [152]. The above studies showed that health status may influence taste and food-related behavior.

On the other hand, there has been increasing evidence that genetic variants in the taste receptor gene may also predispose to changes in health status, suggesting a bidirectional relationship. Given that taste receptors are widely expressed in extraoral tissues, the influence of genetic variation in these genes may be particularly important. In this context, Jiang et al. reported, in a study of the Han Chinese population, that carriers of *TAS2R38* rs77730028 and *TAS2R42* rs1669424 SNVs are at risk of developing chronic rhinitis. At the same time, *TAS2R1* rs385 SNV works protectively, particularly in females [153]. Lee et al. confirmed *TAS2R38* expression in human sinonasal ciliated epithelial cells and proposed its role in mucosal innate defense against upper airway infections [154]. Adappa et al. conducted a study in a group of recalcitrant chronic rhinosinusitis patients undergoing functional endoscopic sinus surgery. They found that allergies, asthma, nasal polyposis, and aspirin sensitivity seem to appear rarely in patients with a particular *TAS2R38* polymorphism [155]. In another study, Xu et al. analyzed single-nucleotide variants SNVs associated with 139 dietary taste preferences (data from GWAS results provided by May-Wilson S et al.) [55] in terms of associations with ovarian insufficiency [156]. It was found that preferences for sweet foods such as cake and white wine were positively associated with the risk of ovarian insufficiency. In contrast, preferences for bitter foods, mackerel, gherkins, cream, and soya milk were negatively associated with the risk of ovarian insufficiency. The authors note in the study that food preferences are a heritable trait and that alterations in taste-related receptor genes may influence ovarian function [156]. Another study on health status indicated a relationship between *TAS2R38* genetic variants and longevity. Melis et al. have demonstrated that the centenarian population from the Sardinia Blue Zone region has an increased frequency of homozygotes carrying the PAV/PAV variant of *TAS2R38* [22]. Recently, Melis et al. conducted another study, including genotyping for *TAS1R2*, *TAS1R3*, *TAS2R38*, and *CD36*, in relation to BMI and sex in near-centenarian individuals and a control cohort, and found distinct distributions of alleles and genotypes for these taste receptor-encoding genes. Authors observed that *TAS1R3* CC rs307355 (C/T), *TAS2R38* PAV/PAV, and rs1761667 (G/A) SNV in *CD36* AA genotypes were most frequently observed in the centenarian population and may have contributed to favorable phenotypes. Authors concluded that the role of taste receptors may be more complex than taste perception and may affect systemic physiology-related processes [157].

Taken together, the available evidence suggests a bidirectional interplay among health status, genetic variation in taste receptor genes, and taste perception, taste preferences, and food preferences, with each potentially influencing the others.

## 7. Conclusions

Taste perception, taste preferences, and food preferences are complex and interrelated traits. Current evidence indicates that genetic variability plays an important role in shaping these phenotypes, with numerous SNVs identified in genes involved in taste transduction, sensory signaling, metabolism, and reward pathways. However, genetic factors alone do not fully explain food-related behaviors, which arise from dynamic interaction between biological, environmental, and health-related influences. Importantly, the relationship between taste-related traits and health status seems to be bidirectional. While, genetic variation may influence taste responsiveness, dietary choices, and susceptibility to certain health outcomes, physiological and pathological conditions may themselves modify taste perception, taste preferences, and food preferences. These findings highlight the complexity of the mechanisms underlying food-related behaviors and emphasize the need to consider both genetic predisposition and health status when investigating taste-related phenotypes.

A better understanding of the genetic architecture underlying taste-related traits may contribute to the development of more personalized nutritional strategies and improve our understanding of dietary choices and eating behaviors. Future research should focus on integrating genetic, phenotypic, environmental, and health-related data using large, well-characterized populations and standardized assessment methods. Such approaches may help clarify the mechanisms linking genetic variation to taste-related behaviors and advance precision nutrition.

## Figures and Tables

**Table 1:** Taste receptors and corresponding tastants.

Taste	Tastant	Taste Receptors	Functional Class	Reference
Sweet	Sucrose, glucose, fructose, saccharin, aspartame, sucralose	TAS1R2/TAS1R3	GPCR	[1,4,30,47]
Umami	monosodium glutamate (MSG), L-glutamate-IMP (inosine monophosphate, guanosine monophosphate, monopotassium glutamate, L-alanine	TAS1R1/TAS1R3	GPCR	[4,34,47,48]
Bitter	Quinine, caffeine, 6-n-propylthiouracil (PROP), phenylthiocarbamide (PTC), stevioside	TAS2R family (e.g., TAS2R4, TAS2R10, TAS2R14, TAS2R38, TAS2R43, TAS2R46)	GPCR	[1,2,4,49,50,51]
Salty	NaCL, Na-gluconate, Na-acetate, Na-citrate	ENaC (subunits SCNN1A/SCNN1B/SCNN1G)	Ion channel	[1,4,30,48]
Sour	Citric acid, acetic acid, lactic acid, ammonium chloride	OTOP1	Ion channel	[3,30,35,52]
Fatty (oleogustus)	Oleic acid, stearic acid, linoleic acid, alpha-linoleic acid	CD36, FFAR4	Lipid sensor	[7,45,46,53]

**Table 2:** Sweet taste. Functional and phenotypic annotation of genetic variants associated with taste and food preferences.

Locus	rsID	Alleles	Consequence	MAF	Associated Trait
*GNAT3*	rs10230573	A/G/T major G	Intron variant	NA	Taste preferences [65]
*TAS1R2*	rs9988418	C/T major C	Missense variant	0.01	Taste preferences [65]
*TAS1R3*	rs35424002	G/A major G	3′ UTR variant	0.13	Taste preferences [65]
*TAS1R2*	rs3935570	G/A/C/T major G	Intron variant	0.25	Taste preferences [58]
*TAS1R2*	rs12033832	G/A/C major G	Synonymous variant	0.33	Taste perception [58] Food preferences [58,61]
*TAS1R2*	rs35874116	T/C major T	Missense variant	0.42	Food preferences [59,60,61,63]
*TAS1R3*	rs307355	T/A/C major C	Intergenic variant	0.49	Taste perception [62]
*TAS1R3*	rs35744813	T/C major C	Intergenic upstream variant	0.23	Taste perception [62]
*TAS2R38*	rs713598 A49P	C/A/G/T major C	Missense variant	0.50	Food preferences [64]
*TAS2R38*	rs1726866 V262A	G/A major A	Missense variant	0.50	Taste perception [67]
*TAS2R38*	rs10246939 I269V	T/C major T	Missense variant	0.45	Taste perception [67]

Data sources: Ensembl, dbSNP, and gnomAD databases. The major allele was derived from European populations. Abbreviations: MAF, minor allele frequency; rsID, reference SNP cluster identifier; NA; not available; AP, Alanine/Proline; VA, Valine/Alanine; IV, Isoleucine/Valine. Note: To maintain structural clarity, diverse behavioral and psychological endpoints extracted from the literature are synthesized into three overarching categories: Taste Perception (sub-classifying studies on taste sensitivity), Taste Preferences (sub-classifying studies on liking), and Food Preferences (sub-classifying studies on cravings, acute food intake, dietary habits, and longitudinal consumption patterns).

**Table 3:** Umami taste. Functional and phenotypic annotation of genetic variants associated with taste and food preferences.

Locus	rsID	Alleles	Consequence	MAF	Associated Trait
*TAS1R3*	rs307377	T/A/C/G major C	Missense variant	0.15	Taste perception [68]
*TAS1R3*	rs35744813	T/C major C	Intergenic upstream variant	0.23	Taste perception [70]
*CD36*	rs1761667	G/A major A	Intron variant	0.50	Taste perception [69] Taste preferences [69] Food preferences [69]
*TAS1R3*	rs307355	T/A/C major C	Intergenic variant	0.49	Taste perception [72]
*TAS1R1*	rs34160967	G/A/C major G	Missense variant	0.41	Taste perception [68,70,72]
*TAS1R1*	rs41278020	C/T major C	Missense variant	0.05	Taste perception [70]
*TAS2R16*	rs846672	G/A major A	Non-coding variant	0.35	Food preferences [71]

Data sources: Ensembl, dbSNP, and gnomAD databases. The major allele was derived from European populations. Abbreviations: MAF, minor allele frequency; rsID, reference SNP cluster identifier. Note: To maintain structural clarity, diverse behavioral and psychological endpoints extracted from the literature are synthesized into three overarching categories: Taste Perception (sub-classifying studies on taste sensitivity), Taste Preferences (sub-classifying studies on liking), and Food Preferences (sub-classifying studies on cravings, acute food intake, dietary habits, and longitudinal consumption patterns).

**Table 4:** Bitter taste. Functional and phenotypic annotation of genetic variants associated with taste and food preferences.

Locus	rsID	Alleles	Consequence	MAF	Associated Trait
*TAS2R38*	rs713598 AP	C/A/G/T major C	Missense variant	0.50	Taste perception [73] Food preferences [24,38,56]
*TAS2R38*	rs1726866 VA	G/A major A	Missense variant	0.50	Taste perception [73] Food preferences [24,38,56]
*TAS2R38*	rs10246939 IV	T/C major T	Missense variant	0.45	Taste perception [73] Food preferences [24,38,56]
*TAS1R3*	rs307355	T/A/C major C	Intergenic variant	0.49	Taste perception [71]
*TAS1R2*	rs9701796	G/A/C major C	Missense variant	0.36	Taste perception [71]
*TAS2R16*	rs860170	C/T major T	Missense variant	0.50	Taste perception [24]
*TAS2R16*	rs846672	A/C/T major C	Intergenic variant	0.50	Food preferences [78]
*TAS2R16*	rs1308724	G/A/C/T major G	Intergenic variant	0.49	Food preferences [78]
*TAS2R19*	rs10772420	G/A major G/A	Missense variant	0.50	Taste perception [78] Taste preferences [78]
*TAS2R43*	rs71443637	T/C major C	Missense variant	0.50	Taste perception [79] Taste preferences [79] Food preferences [79]
*TAS2R31*	rs10772423	C/T major C	Missense variant	0.49	Taste perception [80]
*TAS2R4*	rs2234001	G/A/C/T major G	Missense variant	0.50	Taste perception [24,78] Food preferences [24]
*TAS2R5*	rs2227264	G/A/T| major G	Missense variant	0.49	Taste perception [78]
*TAS2R5*	rs2234012	A/G major A	5′ prime UTR variant	0.49	Taste perception [78]
*TAS2R9*	rs3741845	A/G major G	Missense variant	0.48	Taste perception [80]

Data sources: Ensembl, dbSNP, and gnomAD databases. The major allele was derived from European populations. Abbreviations: MAF, minor allele frequency; rsID, reference SNP cluster identifier; AP, Alanine/Proline; VA, Valine/Alanine; IV, Isoleucine/Valine. Note: To maintain structural clarity, diverse behavioral and psychological endpoints extracted from the literature are synthesized into three overarching categories: Taste Perception (sub-classifying studies on taste sensitivity), Taste Preferences (sub-classifying studies on liking), and Food Preferences (sub-classifying studies on cravings, acute food intake, dietary habits, and longitudinal consumption patterns).

**Table 5:** Salty taste. Functional and phenotypic annotation of genetic variants associated with taste and food preferences.

Locus	rsID	Alleles	Consequence	MAF	Associated Trait
*SCNN1B*	rs239345	T/A major T	Intron variant	0.27	Taste perception [81] Food preferences [82]
*SCNN1B*	rs3785368	G/A major G	Intron variant	0.18	Taste perception [81]
*TAS1R1*	rs34160967	G/A/C major G	Missense variant	0.13	Taste perception [66]
*TAS1R1*	rs17492553	C/A/G/T major T	Splice polypyrimidyne tract variant	0.49	Taste perception [66]
*TAS2R38*	rs713598 AP	C/A/G/T major C	Missense variant	0.50	Taste perception [84]
*TAS2R38*	rs1726866 VA	G/A major A	Missense variant	0.50	Taste perception [84]
*TAS2R38*	rs10246939 IV	T/C major T	Missense variant	0.46	Taste perception [84]

Data sources: Ensembl, dbSNP, and gnomAD databases. The major allele was derived from European populations. Abbreviations: MAF, minor allele frequency; rsID, reference SNP cluster identifier; AP, Alanine/Proline; VA, Valine/Alanine; IV, Isoleucine/Valine. Note: To maintain structural clarity, diverse behavioral and psychological endpoints extracted from the literature are synthesized into overarching categories: Taste Perception (sub-classifying studies on taste sensitivity), and Food Preferences (sub-classifying studies on cravings, acute food intake, dietary habits, and longitudinal consumption patterns).

**Table 6:** Fatty taste. Functional and phenotypic annotation of genetic variants associated with taste and food preferences.

Locus	rsID	Alleles	Consequence	MAF	Associated Trait
*CD36*	rs1761667	G/A major A	Intron variant	0.50	Taste perception [22,45,53,87,88] Taste preferences [69] Food preferences [69,88,89,90]
*CD36*	rs1527483	G/A major G	Intron variant	0.10	Taste perception [88,89] Taste preferences [88,89] Food preferences [88,90]

Data sources: Ensembl, dbSNP, and gnomAD databases. The major allele was derived from European populations. Abbreviations: MAF, minor allele frequency; rsID, reference SNP cluster identifier. Note: To maintain structural clarity, diverse behavioral and psychological endpoints extracted from the literature are synthesized into three overarching categories: Taste Perception (sub-classifying studies on taste sensitivity), Taste Preferences (sub-classifying studies on liking), and Food Preferences (sub-classifying studies on cravings, acute food intake, dietary habits, and longitudinal consumption patterns).

## Data Availability

No new data were created or analyzed in this study. Data sharing is not applicable to this article.

## Supplementary Materials

The following supporting information can be downloaded at: https://www.mdpi.com/article/10.3390/nu18152531/s1, Figure S1. Expression of selected taste receptor genes: *TAS1R1*, *TAS1R2*, *TAS1R3* across selected extraoral tissues; Figure S2. Expression of selected taste receptor genes: *TAS2R38, TAS2R3*, *TAS2R4* across selected extraoral tissues; Figure S3. Expression of selected taste receptor genes: *SCNN1B*, *OTOP1*, *CD36* across selected extraoral tissues.
