# Supplementary material for: Genetic Variants Influencing Taste Perception and Food Preferences: Current Evidence
Source: Nutrients. 2026 Aug 4;18(15):2531. doi: 10.3390/nu18152531 (PMC13468555; doi:10.3390/nu18152531)
Supplement: Supplementary file 1 [file nutrients-18-02531-s001.zip › nutrients-4441113-supplementary.pdf]

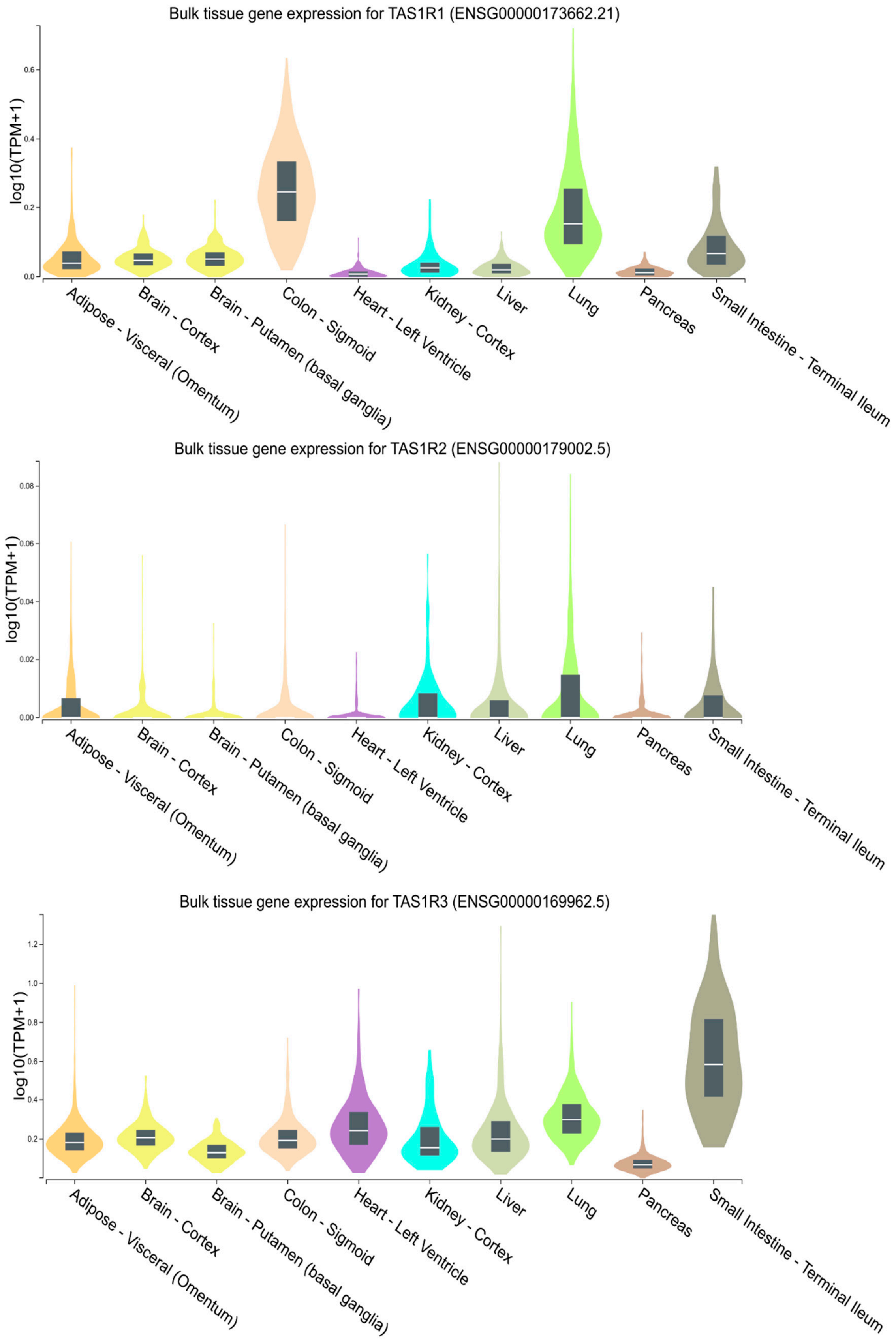

Figure S1. Expression of selected taste receptor genes: *TAS1R1, TAS1R2, TAS1R3* across selected extraoral tissues. GTEx-generated violin plots of taste receptor gene expression across selected

extraoral tissues (SVG export). Data Source: GTEx Analysis Release V10 (dbGaP Accession phs000424.v10.p2).

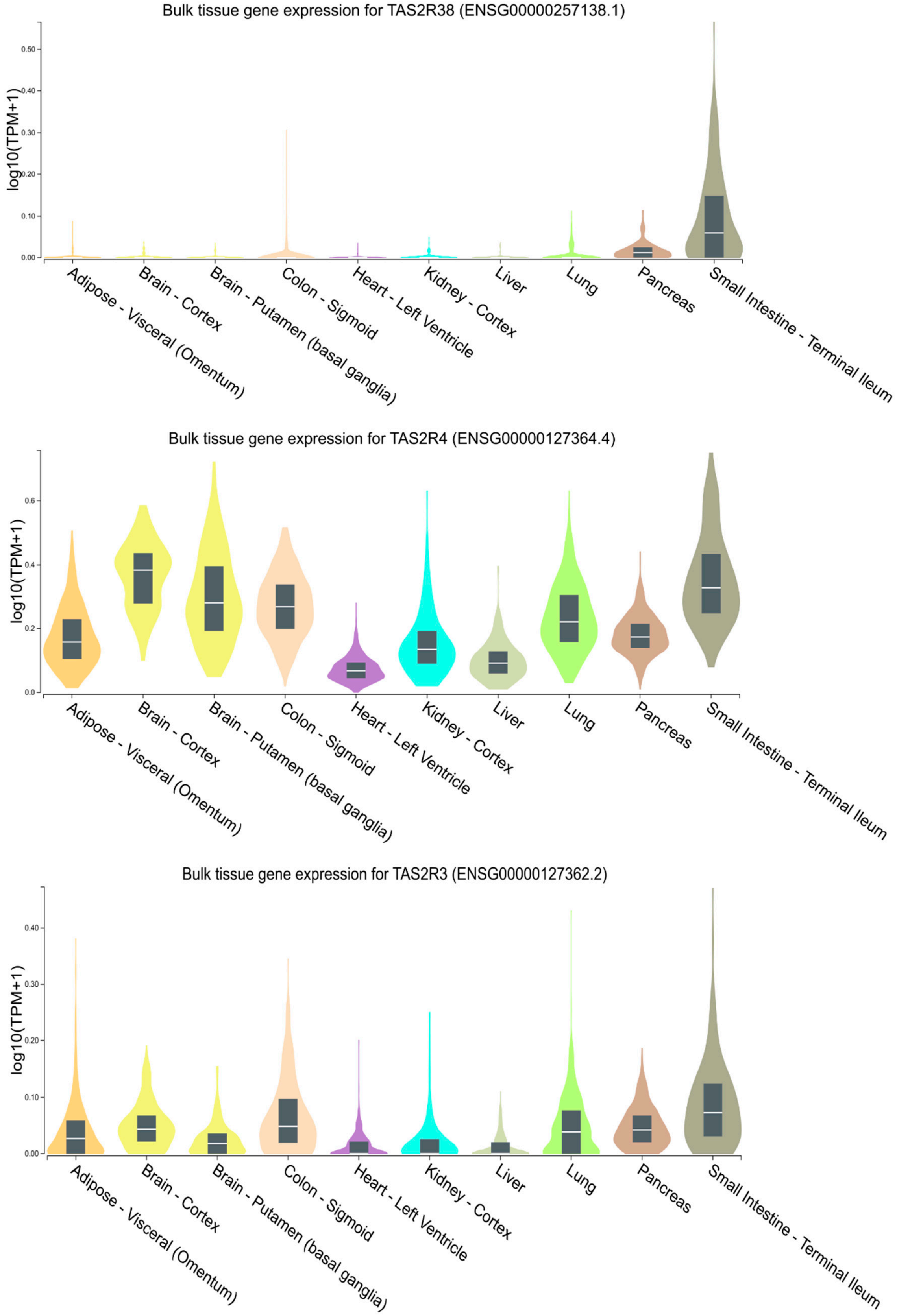

Figure S2. Expression of selected taste receptor genes: *TAS2R38, TAS2R3, TAS2R4* across selected

extraoral tissues. GTEx-generated violin plots of taste receptor gene expression across selected extraoral tissues (SVG export). Data Source: GTEx Analysis Release V10 (dbGaP Accession phs000424.v10.p2).

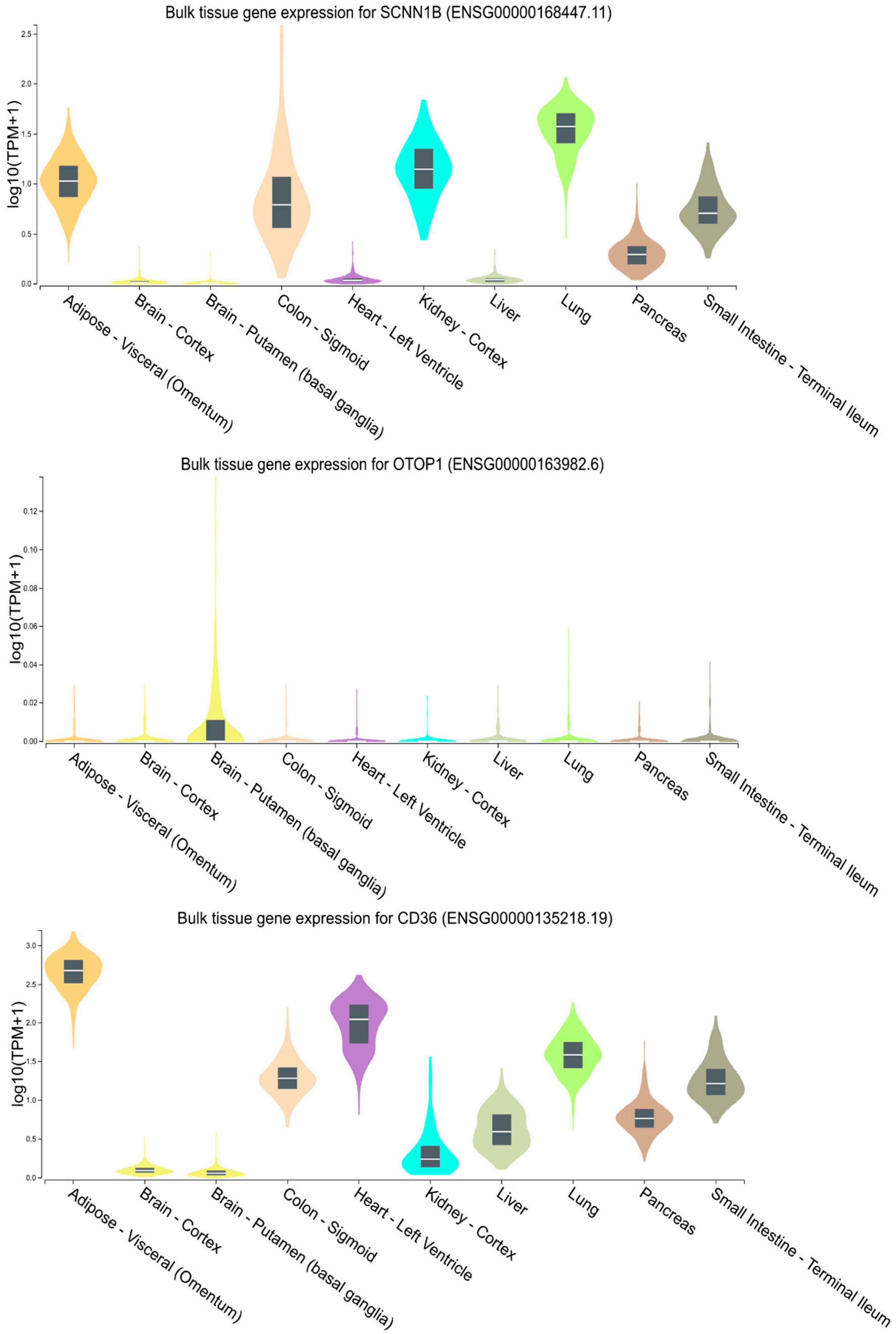

Figure S3. Expression of selected taste receptor genes: *SCNN1B*, *OTOP1*, *CD36* across selected extraoral

tissues. GTEx-generated violin plots of taste receptor gene expression across selected extraoral tissues (SVG export). Data Source: GTEx Analysis Release V10 (dbGaP Accession phs000424.v10.p2).
